# Supplementary material for: Methionine-triggered growth arrest reveals activation of Gcn2 by methionine transporter endocytosis
Source: bioRxiv. 2025 May 14:2025.05.12.653625. Preprint. [Version 1] doi: 10.1101/2025.05.12.653625 (PMC12132241; doi:10.1101/2025.05.12.653625)

bioRxiv preprint doi: https://doi.org/10.1101/2025.05.12.653625; this version posted May 14, 2025. The copyright holder for this preprint (which was not certified by peer review) is the author/funder, who has granted bioRxiv a license to display the preprint in perpetuity. It is made available under aCC-BY 4.0 International license.

Supplementary Figure Legends

Figure S1. Specific amino acids inhibit septin assembly.

(A) Yeast cells expressing Shs1-mCherry (red, septin ring) were analyzed by fluorescence microscopy and overlayed with DIC images.

(B) Quantification of the results shown in (A) over three biological replicate experiments (n=3).

(C) Yeast cells expressing Shs1-FLAG (chromosomally tagged) were cultured and stimulated with the indicated amino acids. Cell lysates were analyzed by SDS-PAGE and immunoblotting was performed for using α-FLAG antibodies and α-G6PDH antibodies as a loading control.

Figure S2. Activation of mitophagy by amino acid supplementation.

For individual cells expressing Vph1-mNG (vacuole membrane) and Tom70-mCherry (mitochondria), mean fluorescence in the vacuole was measured for the indicated time points and conditions (n≥30 cells).

Figure S3. Features of Met-triggered mitophagy.

(A) For individual cells expressing Vph1-mNG (vacuole membrane) and Tom70-mCherry (mitochondria), mean fluorescence in the vacuole was measured for the indicated methionine concentrations (n≥30 cells per condition).

(B) For individual cells expressing Vph1-mNG (vacuole membrane) and Tom70-mCherry (mitochondria), mean fluorescence in the vacuole was measured for the indicated time points and conditions (n≥30 cells).

bioRxiv preprint doi: https://doi.org/10.1101/2025.05.12.653625; this version posted May 14, 2025. The copyright holder for this preprint (which was not certified by peer review) is the author/funder, who has granted bioRxiv a license to display the preprint in perpetuity. It is made available under aCC-BY 4.0 International license.

Figure S4. Tco89-dependent activation of mitophagy by Met supplementation.

(A) Cells expressing Vph1-mTagBFP2 (vacuole membrane, blue) and Tom70-mCherry (mitochondria, red) were imaged by fluorescence microscopy and DIC (overlayed).

(B) For individual cells from the experiment shown in (A), mean Tom70-mCherry fluorescence in the vacuole was measured for the indicated time points and conditions (n≥30 cells).

(C) Cells expressing Vph1-mTagBFP2 (vacuole membrane, blue) and Tom70-mCherry (mitochondria, red) were imaged by fluorescence microscopy and DIC (overlayed).

(D) For individual cells from the experiment shown in (A), mean Tom70-mCherry fluorescence in the vacuole was measured for the indicated time points and conditions (n≥30 cells).

Figure S5. Met-triggered mitophagy is dependent on the Gcn2 pathway.

(A) For individual cells expressing Vph1-mNG (vacuole membrane) and Tom70-mCherry (mitochondria), mean fluorescence in the vacuole was measured for the indicated time points and conditions (n≥30 cells).

(B) For individual cells expressing Vph1-mNG (vacuole membrane) and Tom70-mCherry (mitochondria), mean fluorescence in the vacuole was measured for the indicated time points and conditions (n≥30 cells).

Figure S6. Ppz phosphatases are required for Met-triggered Gcn2 activation.

(A) Analysis of yeast overnight growth with the indicated culture conditions (n≥3).

(B) Cells cultured in standard media (SCD) or with the indicated treatment were collected and cell lysates were analyzed by SDS-PAGE and immunoblotting for pRPS6 as a readout for TORC1 activity. G6PDH was used as a loading control.

(C) Cells harboring chromosomal FLAG-tagged Gcn4 were cultured in standard media (SCD) or with the indicated treatment were collected and cell lysates were analyzed by SDS-

bioRxiv preprint doi: https://doi.org/10.1101/2025.05.12.653625; this version posted May 14, 2025. The copyright holder for this preprint (which was not certified by peer review) is the author/funder, who has granted bioRxiv a license to display the preprint in perpetuity. It is made available under aCC-BY 4.0 International license.

PAGE and immunoblotting for Gcn4-FLAG expression as a readout for Gcn2 activity. G6PDH was used as a loading control.

(D) Quantification of immunoblotting results shown in (C) from three biological replicate experiments (n=3).

bioRxiv preprint doi: https://doi.org/10.1101/2025.05.12.653625; this version posted May 14, 2025. The copyright holder for this preprint (which was not certified by peer review) is the author/funder, who has granted bioRxiv a license to display the preprint in perpetuity. It is made available under aCC-BY 4.0 International license.

Supplemental Figure S1

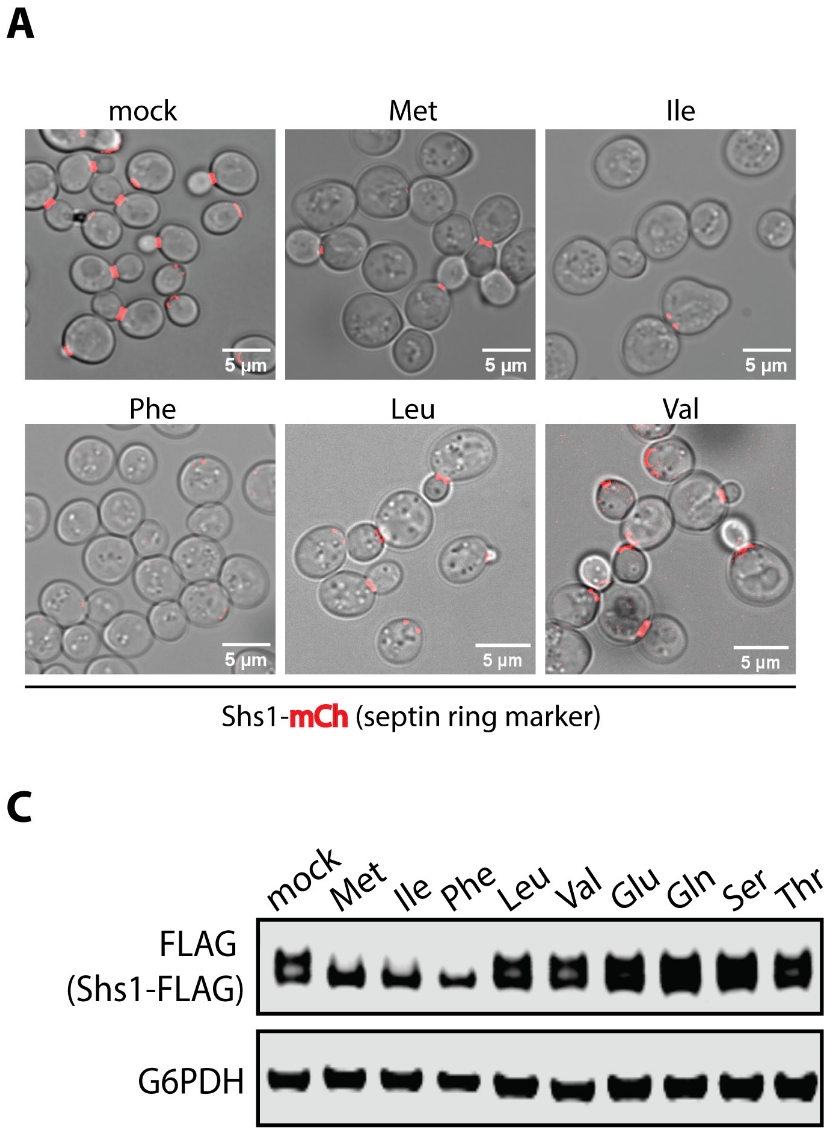

bioRxiv preprint doi: https://doi.org/10.1101/2025.05.12.653625; this version posted May 14, 2025. The copyright holder for this preprint (which was not certified by peer review) is the author/funder, who has granted bioRxiv a license to display the preprint in perpetuity. It is made available under aCC-BY 4.0 International license.

Supplemental Figure S2

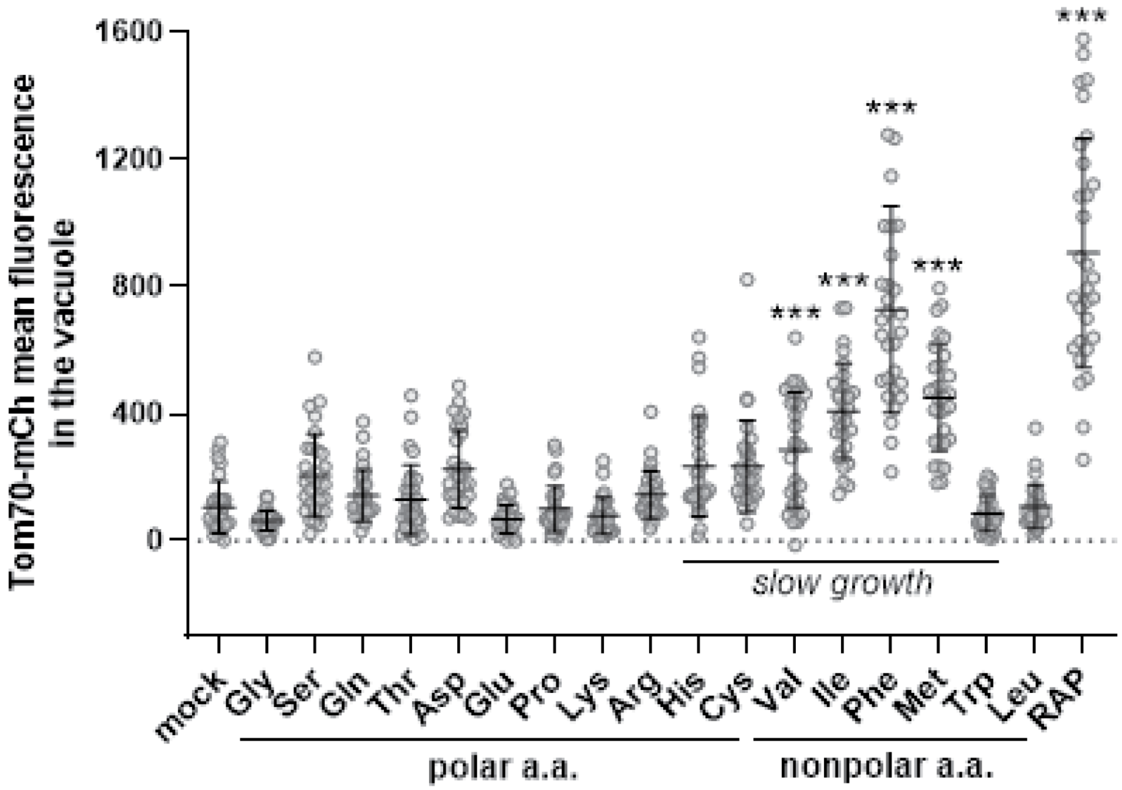

bioRxiv preprint doi: https://doi.org/10.1101/2025.05.12.653625; this version posted May 14, 2025. The copyright holder for this preprint (which was not certified by peer review) is the author/funder, who has granted bioRxiv a license to display the preprint in perpetuity. It is made available under aCC-BY 4.0 International license.

Supplemental Figure S3

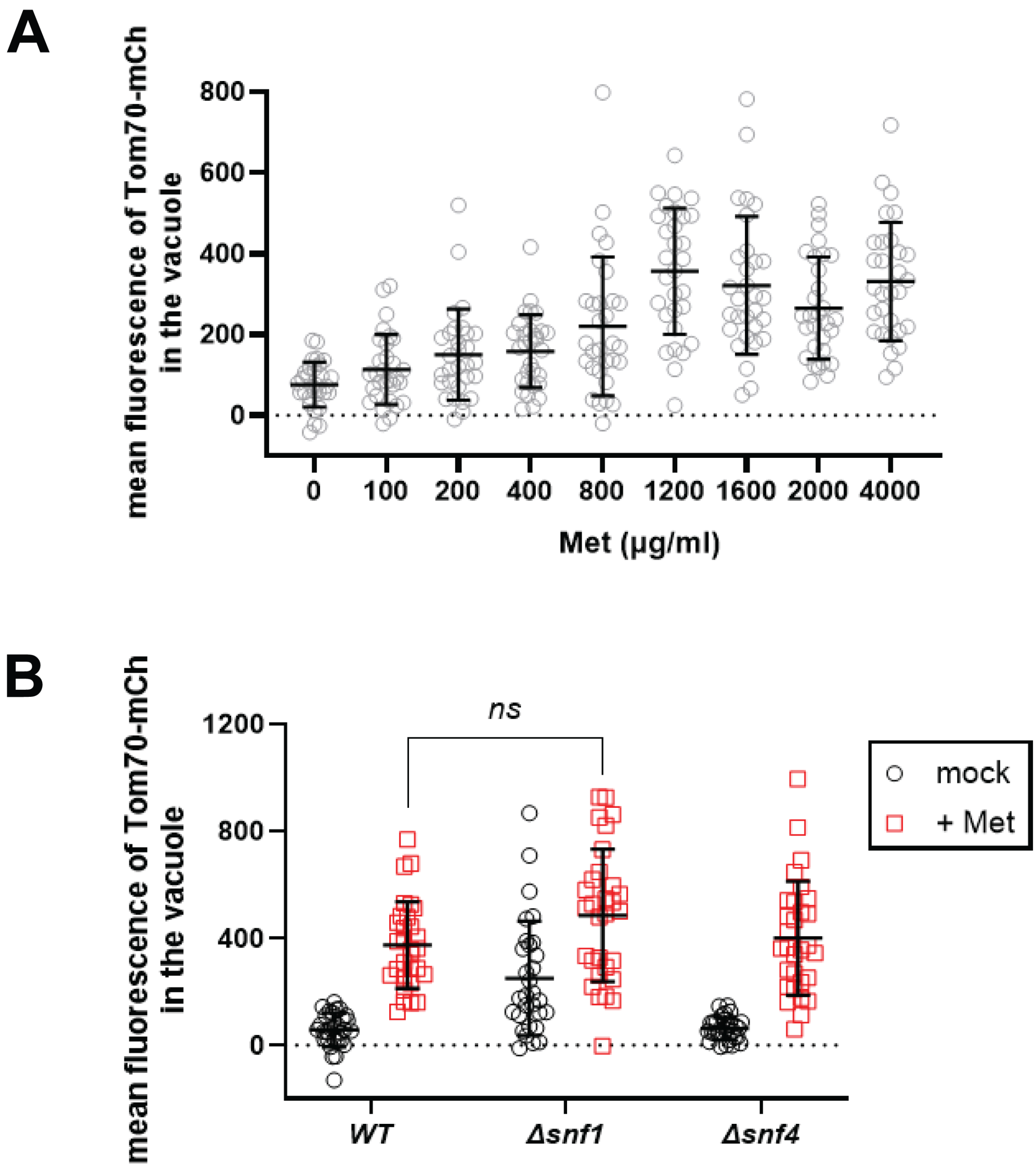

bioRxiv preprint doi: https://doi.org/10.1101/2025.05.12.653625; this version posted May 14, 2025. The copyright holder for this preprint (which was not certified by peer review) is the author/funder, who has granted bioRxiv a license to display the preprint in perpetuity. It is made available under aCC-BY 4.0 International license.

Supplemental Figure S4

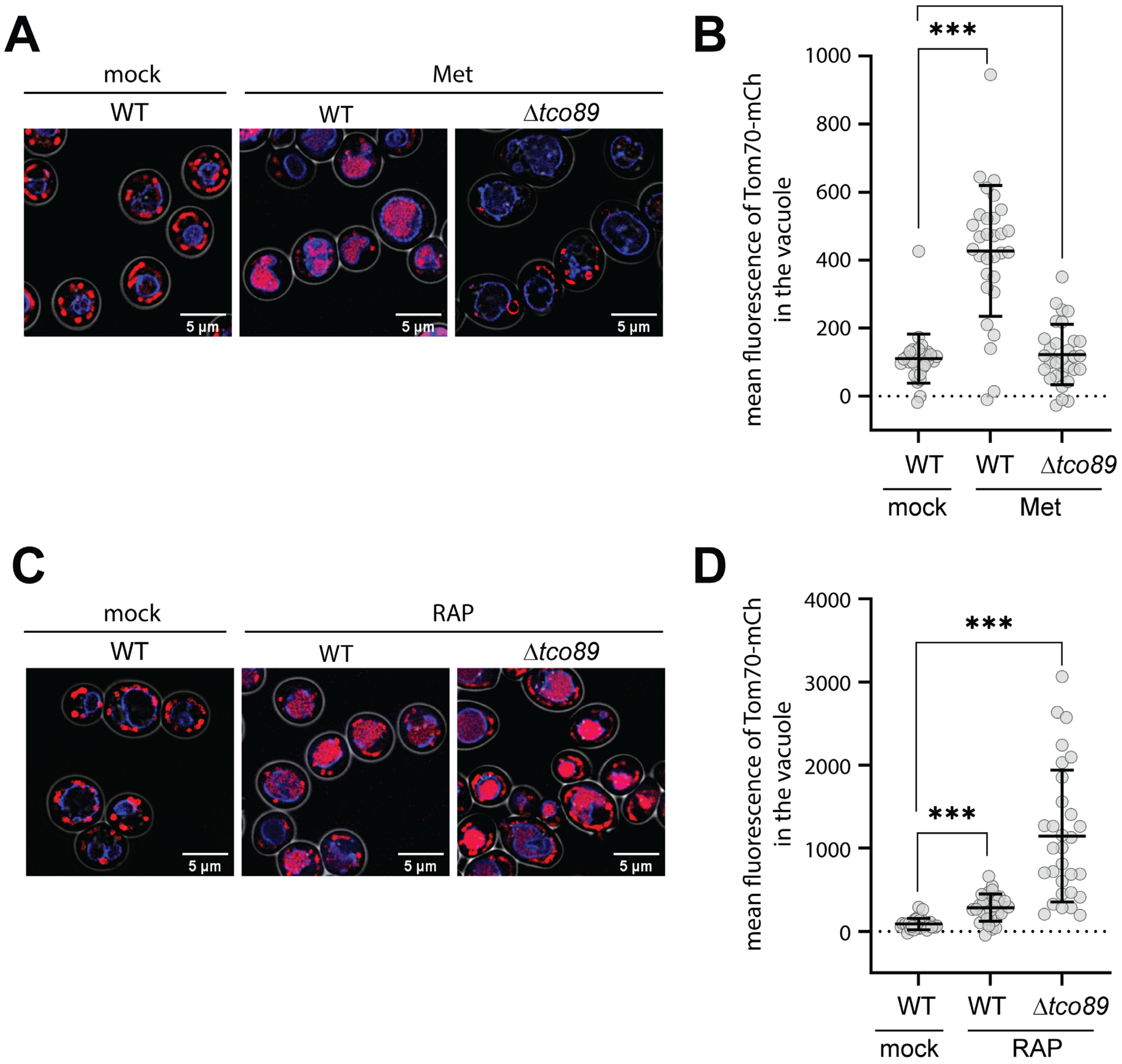

bioRxiv preprint doi: https://doi.org/10.1101/2025.05.12.653625; this version posted May 14, 2025. The copyright holder for this preprint (which was not certified by peer review) is the author/funder, who has granted bioRxiv a license to display the preprint in perpetuity. It is made available under aCC-BY 4.0 International license.

Supplemental Figure S5

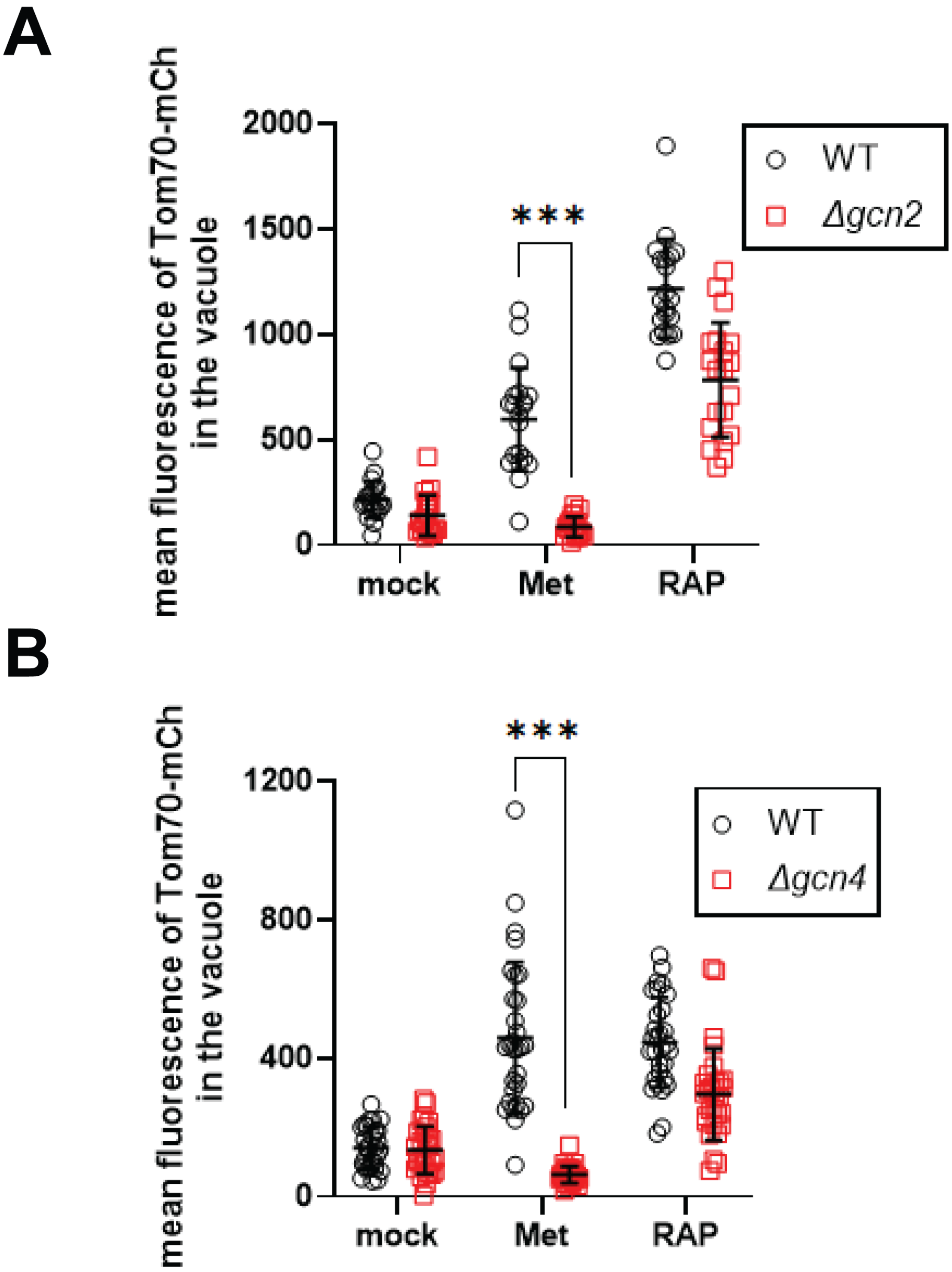

bioRxiv preprint doi: https://doi.org/10.1101/2025.05.12.653625; this version posted May 14, 2025. The copyright holder for this preprint (which was not certified by peer review) is the author/funder, who has granted bioRxiv a license to display the preprint in perpetuity. It is made available under aCC-BY 4.0 International license.

Supplemental Figure S6

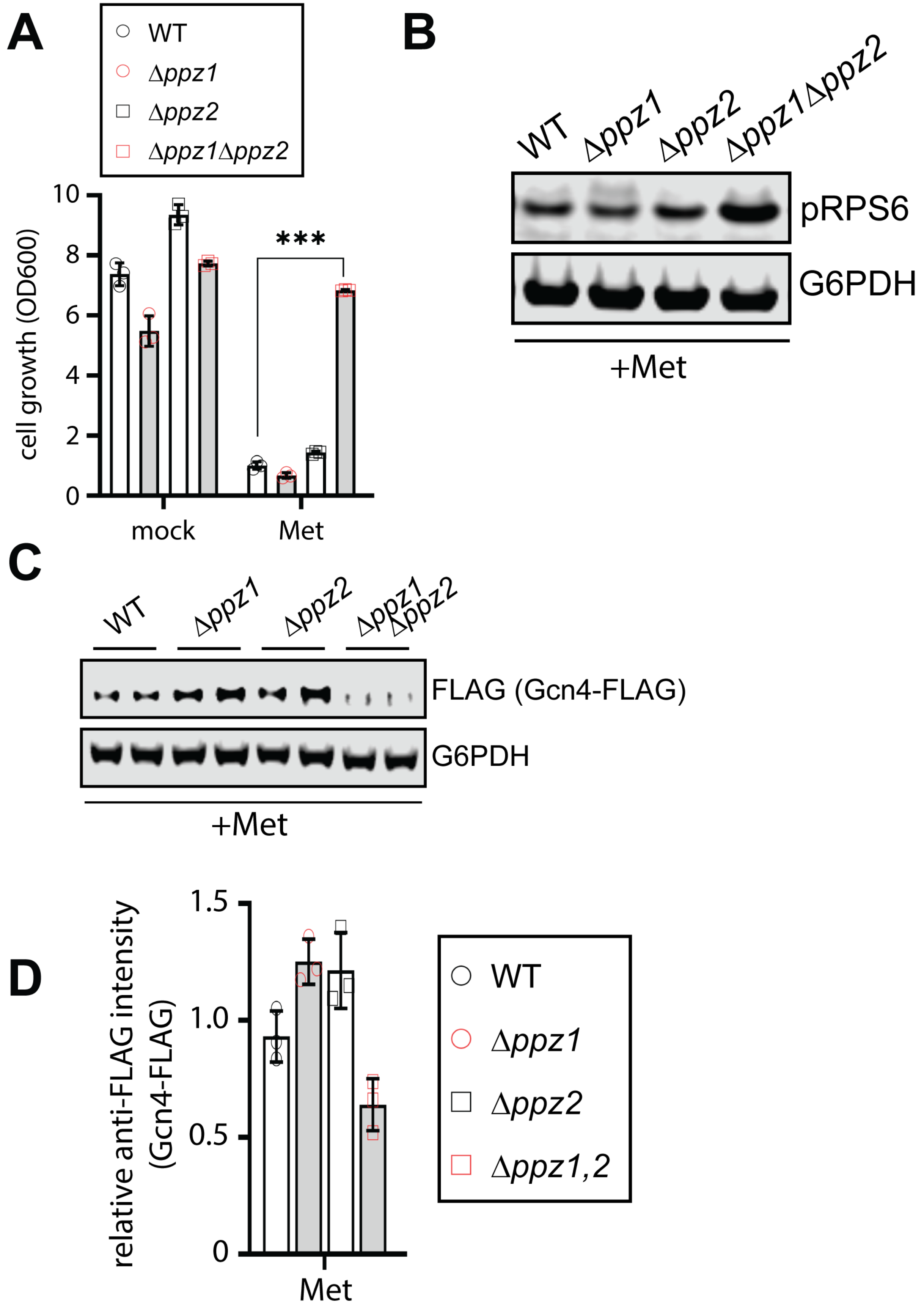

Supplement: Supplement 1 [file NIHPP2025.05.12.653625v1-supplement-1.pdf]
